# Prediagnosis Depression Rather Than Anxiety Symptoms Is Associated with Decreased Ovarian Cancer Survival: Findings from the Ovarian Cancer Follow-Up Study (OOPS)

**DOI:** 10.3390/jcm11247394

**Published:** 2022-12-13

**Authors:** Yi-Zi Li, Xue Qin, Fang-Hua Liu, Wen-Xiao Chen, Yi-Fan Wei, Na Wang, Shi Yan, Ye Kang, Yu-Hong Zhao, Song Gao, Ting-Ting Gong, Qi-Jun Wu

**Affiliations:** 1 Department of Clinical Epidemiology, Shengjing Hospital of China Medical University, Shenyang 110004, China; 2 Clinical Research Center, Shengjing Hospital of China Medical University, Shenyang 110004, China; 3 Liaoning Key Laboratory of Precision Medical Research on Major Chronic Disease, Shengjing Hospital of China Medical University, Shenyang 110004, China; 4 Department of Obstetrics and Gynecology, Shengjing Hospital of China Medical University, Shenyang 110004, China; 5 Department of Sports Medicine and Joint Surgery, The People’s Hospital of Liaoning Province, Shenyang 110000, China; 6 Department of Epidemiology, School of Public Health, Fudan University, Shanghai 200433, China; 7 Department of Pathology, Shengjing Hospital of China Medical University, Shenyang 110004, China; 8 Key Laboratory of Reproductive and Genetic Medicine, National Health Commission, China Medical University, Shenyang 110004, China

**Keywords:** anxiety, cohort, depression, ovarian cancer, survival

## Abstract

Background: The relationship between prediagnosis depression, anxiety symptoms, and ovarian cancer (OC) survival is unknown. We aimed to explore these associations to provide further epidemiological evidence. Methods: We investigated the relationship between prediagnosis depression, anxiety symptoms, and OC survival in a prospective cohort study of newly diagnosed OC patients aged 18–79 years. Depression and anxiety symptoms were assessed using the Patient Health Questionnaire 9 and Generalized Anxiety Disorder 7 at diagnosis, respectively. Deaths were ascertained until 31 March 2021 via medical records and active follow-up. Multivariable-adjusted Cox proportional hazards regression was used to estimate hazard ratios (HRs) and 95% confidence intervals (CIs) with prediagnosis depression and anxiety symptoms and all-cause mortality of OC. Results: We found 56 (9.4%) and 235 (39.3%) OC patients with depression and anxiety symptoms, respectively. During a median follow-up of 37.2 months (interquartile range 24.7–50.2 months), 130 deaths were confirmed. Compared with non-depression symptoms, patients with prediagnosis depressive symptoms showed a significantly increased risk of OC mortality (HR = 2.10, 95% CI: 1.20–3.70). Of note, the association was still robust when focusing on the OC patients with severe depressive symptoms (HR = 2.10, 95% CI: 1.07–4.12). However, we observed no association between prediagnosis anxiety symptoms of different severity and OC mortality. Interestingly, OC patients with combined moderate depression and anxiety symptoms had a significantly increased risk of OC mortality (HR = 3.23, 95% CI: 1.14–9.11) compared to those with no symptoms of depression and anxiety. Notably, Wilms’s tumor 1 was significantly associated with depression and anxiety symptoms (*p* < 0.05). Conclusions: Prediagnosis depression increases the risk of OC mortality. Large multicenter studies are required to confirm this finding.

## 1. Introduction

Ovarian cancer (OC) is the deadliest gynecologic cancer and the fourth-leading cause of cancer-related death in women [1]. In 2020, an estimated 313,959 women were diagnosed with OC and 207,252 died from this condition worldwide [1]. Due to the lack of early and specific symptoms, more than 75% of OC cases are diagnosed at an advanced stage, with an age-standardized survival rate of five years below 25% [1,2]. Although it has been proven that cancer clinical characteristics and treatment are important determinants of OC prognosis, evidence of the influence of modifiable prediagnosis psychosocial factors is still limited.

During the last decade, depression and anxiety have been proposed as the most common psychosocial characteristics that could play an etiologic role in and bear a prognostic impact on cancers [3,4,5]. Depression and anxiety are prevalent and disabling mental disorders that frequently co-occur with health outcomes [6,7]. Recent evidence suggests that psychosocial mechanisms of depression and anxiety include the expression of stress hormones such as cortisol and catecholamines, respectively, from the deregulated hypothalamic–pituitary–adrenal axis and the sympathetic nervous system, which may promote growth and progression of OC via stress-mediated pathways [8]. Previous evidence has shown that OC patients present a range of psychological symptoms during stages of diagnosis, treatment, and survival, mainly with high levels of depression and anxiety [9]. A recent meta-analysis suggests that the prevalence of depression and anxiety in OC patients is considerably greater across the treatment spectrum than in the healthy female population [10]. Specifically, pretreatment, in-treatment, and posttreatment depression prevalence among OC patients was 25.34%, 22.99%, and 12.71%, respectively [11]. Furthermore, pretreatment, in-treatment, and posttreatment anxiety prevalence among OC patients was 19.12%, 26.23%, and 27.09%, respectively [11]. However, little is known about the relationship between depression and anxiety symptoms and mortality of OC. Recently, only two studies have evaluated the association between prediagnosis depression and anxiety and OC survival [12,13]. Clarke et al. [12] reported that a diagnosis of depression within the year prior to a cancer diagnosis, evaluated by the Elixhauser comorbidity score, was inversely associated with long-term survival based on a retrospective study of 642 high-grade serous OC patients. Additionally, Poole et al. [13] found no association between prediagnosis phobic anxiety symptoms and OC survival utilizing the Crown–Crisp phobic anxiety index based on 779 cases from the Nurses’ Health Studies.

Given the current lack of prospective evidence regarding the role of prediagnosis depression and anxiety symptoms in OC survival, we analyzed data from a prospective cohort study—the Ovarian Cancer Follow-Up Study (OOPS)—conducted in Shenyang, China, to investigate this topic.

## 2. Methods: Study Population

**Figure 1:**
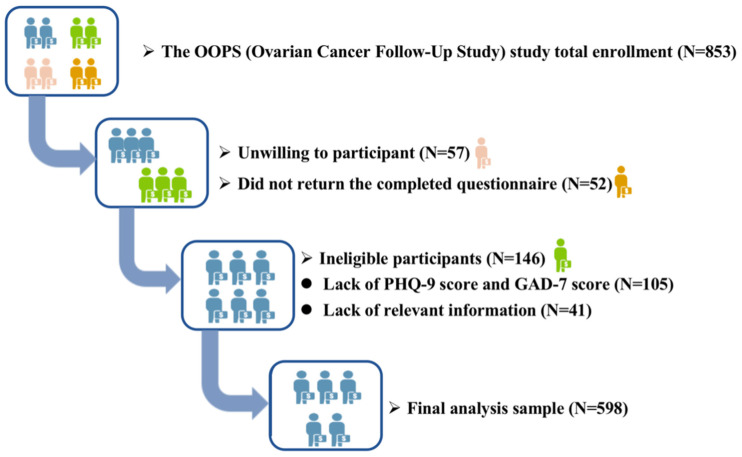
Flow of participants through the Ovarian Cancer Follow-Up Study (OOPS) study.

The OOPS was an ongoing prospective longitudinal cohort study that started in 2015 and consecutively enrolled newly diagnosed OC patients aged 18–75 years. The patients were enrolled from the Gynecological Oncology Ward at Shengjing Hospital of China Medical University, Shenyang, China from 2015 to 2020 [14]. Briefly, 853 OC patients had were recruited and the overall study participation rate exceeded 90% (*n* = 796). For this analysis, we excluded OC patients without completed questionnaires (*n* = 52), with questionnaires containing 11 (10%) or more blank food items (*n* = 24), questionnaires lacking mentally relevant information (*n* = 105), and those with implausible caloric intakes (<500 or >3500 calories/day) (*n* = 17) [14,15]. Finally, 598 OC patients were included in the current analysis (Figure 1). The protocol for OOPS was approved by the Medical Ethics Committee of Shengjing Hospital of China Medical University. All patients provided written informed consent before participation.

At baseline, OC patients completed a detailed self-administered questionnaire of demographics and lifestyle factors, including educational level, monthly household income, physical activity, smoking status, alcohol drinking, and tea drinking [16,17]. Anthropometric measurements, including the current weight, height, and circumference of the waist and hips, were also collected by trained staff following a standard protocol [16,17]. During the in-person interview, newly diagnosed OC patients were asked about their usual frequency of consumption of each food item and sleep status, which were included in the 111-item validated food frequency and the Pittsburgh Sleep Quality Index (PSQI) questionnaire in the 12 months before diagnosis, respectively [17,18,19]. The PSQI consists of 19 items that could reflect on seven major sleep components, including sleep quality and sleep duration, with each component weighted on a 0–3 scale and a maximum score of 21. A total score higher than 5 indicates poor sleep quality [20].

Stressful life events refer to everyday life events, including 10 items in the past 2 years [21]. Based on the electronic medical records of the hospital information system, each OC patient was linked with her clinical information. These characteristics included age at diagnosis, histological type, histopathological grade, International Federation of Gynecology and Obstetrics (FIGO) stage, residual lesions, and comorbidities (hypertension, coronary heart disease, diabetes, and others).

Immunohistochemical (IHC) analysis was carried out to acquire specimens of OC and adjacent tissue after surgery. In brief, specimens were formalin-fixed and paraffin-embedded, then dewaxed and hydrated according to routine methods. Antigen retrieval was done using citrate buffer (pH 6.0 at 92 °C), and endogenous peroxidase activity was blocked with fresh 3% hydrogen peroxide. Afterwards, the sections were blocked with normal serum solution and incubated with a primary antibody against Wilms’s tumor 1 (WT-1), estrogen receptor (ER), progesterone receptor (PR), vimentin, and p53 (1:500, Abcam) at 4 °C overnight. The sections were subsequently incubated with corresponding secondary antibodies for 30 min at 37 °C and rinsed with phosphate-buffered saline (PBS). Finally, sections were treated with diaminobenzidine and hematoxylin for coloration and counterstaining. Staining was evaluated according to positively stained cell percentage and staining intensity. All indicators [WT-1, ER, PR, vimentin, and p53] were divided into positive and negative. IHC expression was manually confirmed by two independent experienced pathologists.

### 2.1. Assessment of Depression and Anxiety

Prediagnosis mental health status was assessed at recruitment with the 9-item Patient Health Questionnaire (PHQ-9) and the 7-item Generalized Anxiety Disorder (GAD-7). The PHQ-9 is a self-report questionnaire comprising nine questions and is used to calculate a depression severity score based on symptoms over a two-week period with high internal consistency, reliability, and validity [22,23]. The PHQ-9 is based on the Primary Care Evaluation of Mental Disorders (PRIME-MD) diagnostic instrument and utilizes nine DSM-IV criteria. Each of the nine items is scored from 0 (not at all) to 3 (nearly every day), giving a total score range between 0 and 27. The total score reflects depression symptom severity with a range of 0–4, 5–9, 10–14, 15–19, and ≥20, indicating no, mild, moderate, moderately severe, and severe depressive symptoms, respectively [24]. In addition, patients with PHQ-9 ≥10 are considered to have symptomatic depression [25]. The GAD-7 is a self-administered tool for assessing generalized anxiety disorder and anxiety-related symptoms on the basic of PRIME-MD, which has good sensitivity and specificity [26,27]. Each item is scored on a scale of 0 to 3, with a total score range between 0 and 21 [28]. Total scores of 5, 10, and 15 indicate mild, moderate, and severe anxiety, respectively. The optimal cutoff score is 5 for anxiety symptoms [29].

### 2.2. Cohort Follow-Up and Outcome Ascertainment

The OOPS participants were followed up until mortality from any cause or the last follow-up (31 March 2021). Data on vital statistics were obtained annually by active follow-up of patients and record linkage to the Vital Statistics Unit in the Liaoning Center for Disease Control and Prevention.

## 3. Statistical Analysis

We compared descriptive statistics of general and clinical characteristics based on prediagnostic depression and anxiety symptoms using analysis of variance for continuous variables and the chi-square test for categorical variables. The Kaplan–Meier technique was used to plot crude survival curves and estimate the overall crude survival (OS) probabilities. Adjusted hazard ratios (HRs) and the corresponding 95% confidence intervals (CIs) were derived from Cox proportional hazards regression models where the entry time was the date at which the OC patients were enrolled in the OOPS. The exit date was when the participant died or was censored due to loss to follow-up or end of study follow-up on 31 March 2021, using whichever censoring date occurred first. The proportional hazards assumption was tested by including an interaction term between depression and anxiety symptoms with the logarithm of time, and no violations were discovered (all *p* > 0.05).

The total depression and anxiety scores were dichotomized, and patients who scored above 10 and 5 were considered as having symptomatic depression and anxiety, respectively. The HR and 95% CI for each category were estimated compared to those with non-depression symptoms and non-anxiety symptoms. Models were adjusted for potential confounding factors. Model 1 included age at diagnosis (continuous, years) and body mass index (BMI) (continuous, kg/m^2^). Model 2 was further adjusted for dietary changes (yes or no), education (junior secondary or below, senior high school/technical secondary school, and junior college/university or above), number of menstrual years (continuous), parity (≤1 or ≥2), and physical activity (continuous, metabolic equivalents of task hours/day). Further adjustments were also made for total sleep duration (continuous, hours/day), sleep quality (good or bad, PSQI score ≤5 defined good, PSQI score >5 defined bad) [19], and stressful life events in the past two years before diagnosis (yes or no) to minimize the impact of lifestyle factors and dietary factors on survival. In model 3, we further adjusted for comorbidities (yes or no), FIGO stage (I–II, III–IV, or unknown), histological type (serous or nonserous), histopathological grade (well, moderate, or poorly differentiated), and residual lesions (none, <1, or ≥1 cm). Multicollinearity among covariance was evaluated using the variance inflation factor. Moreover, we performed a further analysis evaluating whether the severity of depression and anxiety symptoms were associated with OC survival, adjusting in the same multivariate model (model 3).

Stratified analyses were conducted for age at diagnosis (<50 vs. ≥50 years), menopausal status (no vs. yes), FIGO stage (I–II vs. III–IV), residual lesions (no vs. yes), histological type (serous vs. nonserious), BMI (<24 vs. ≥24 kg/m^2^), total sleep duration (<8 vs. ≥8 h/day), sleep quality (bad vs. good), and any major events (no vs. yes). We included multiple interaction terms and tested their significance using a likelihood ratio test to seek out interaction. Analyses were performed using SAS version 9.4 (SAS Institute, Cary, NC, USA). Two-sided *p* values less than 0.05 were considered statistically significant.

## 4. Results

Table 1 shows the baseline characteristics of the participants in the cohort study. During a median follow-up of 37.2 months (interquartile range 24.7–50.2 months), 130 deaths were documented due to all causes. Among all OC patients, the prevalence of depression and anxiety was 9.4% and 39.3%, respectively. OC patients with depression had a lower degree of education and higher parity. They were more likely to have shorter sleep duration and worse sleep quality, whereas patients with anxiety symptoms were more likely to be on a lower income and their sleep status was worse than non-anxiety patients. A nonserous histological subtype, later-stage disease, and more significant residual disease were statistically significantly associated with worse survival in this cohort (Supplementary Table S1).

**Figure 2:**
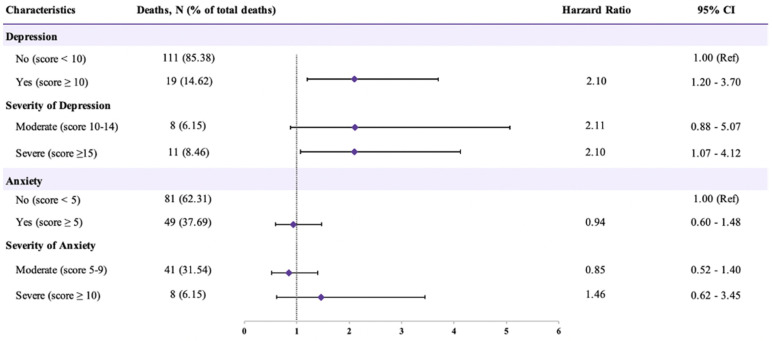
Adjusted hazard ratio and 95% confidence intervals for the association between depression and anxiety symptoms and total mortality among 598 ovarian cancer patients. CI, confidence interval, Ref, reference. Depression symptom was evaluated according to Patient Health Questionnaire-9. Anxiety symptom was evaluated according to Generalized Anxiety Disorder-7.

The association of prediagnosis depression and anxiety symptoms with OS of OC patients is shown in Figure 2, Supplementary Table S2 and Figure S1. Of these 130 deaths, 19 (14.62%) and 11 (8.46%) had depression and severe depression, while 41 (31.54%) and 8 (6.15%) had anxiety and severe anxiety, respectively. In most adjusted models, patients with depressive symptoms had a significantly increased risk of OC mortality than non-depression symptom patients (HR = 2.10, 95% CI: 1.20–3.70). Additionally, worse survival was detected for OC patients with severe depression symptoms when compared with non-depression symptom patients (HR = 2.10, 95% CI: 1.07–4.12). However, neither anxiety symptoms nor severity was associated with OC survival. Notably, OC patients with combined moderate depression and anxiety symptoms had a substantially increased risk of OC mortality (HR = 3.23, 95% CI: 1.14–9.11) compared with no depression and anxiety symptoms (Table 2).

In subgroup analyses, significant associations between depression symptoms and OC mortality were detected in patients with age at diagnosis less than 50 years, BMI < 24 kg/m^2^, sleep duration ≥ 8 h/day, poor sleep quality, no stressful life events, nonserous histological type, residual lesions, and patients with stage Ⅰ–Ⅱ (Supplementary Figures S2 and S3). Additionally, OC patients with anxiety symptoms had an increased mortality risk in the subgroup that had never experienced any stressful life events. No statistically significant interactions were observed, except for the association between depression symptoms and OC mortality stratified by age at diagnosis and histological type (*p* < 0.05). However, the subgroup analyses of immunohistochemical biomarkers showed that significant inverse associations were present in women with depression who were WT-1-negative and vimentin-negative (Supplementary Figures S4 and S5). Notably, there were significant interactions between WT-1 expression and depression and anxiety symptoms (*p* < 0.05).

## 5. Discussion

### 5.1. Main Findings

To our knowledge, this is the first prospective cohort study to investigate the relationship between prediagnosis depression and anxiety symptoms and OC survival, assessed with PHQ-9 and GAD-7, respectively. Our findings revealed that prediagnosis depression, not anxiety symptoms, was associated with decreased OC survival.

### 5.2. Interpretation

Although several previous studies have evaluated the association of depression and anxiety symptoms with OC risk [10], the evidence for the influence of depression and anxiety symptoms on OC survival is scarce and limited. Only one retrospective study of 642 high-grade serous OC patients suggested that depression symptoms, assessed by Elixhauser comorbidity score within the year prior to diagnosis, were inversely associated with OC survival [12]. Although we evaluated the depression symptoms of OC patients with PHQ-9 in a prospective cohort design, our results were consistent with their findings. In support of our findings, evidence from other cancers has linked depressive symptomology with reduced cancer survival [30,31]. For example, a cohort study of 3095 breast cancer patients reported that women with newly developed depression before diagnosis had a modest but significantly increased risk of death [32]. Likewise, in a British cohort study of 2144 head and neck cancer patients, worse survival was evident among patients with pretreatment depression symptoms [33]. For anxiety symptoms, our finding is in line with a previous study of 779 OC patients in the US [13], where a nonsignificant association between prediagnosis symptoms, evaluated by the Crown–Crisp phobic anxiety index, and OC survival was observed.

The results of several previous studies on breast cancer agree with our findings to a certain degree. These studies did not support anxiety symptoms having an important impact on breast cancer outcomes [34,35]. Nevertheless, the prevalence of prediagnosis depression and anxiety in our study is inconsistent with a previous meta-analysis [11], which might be attributed to several factors, including an inability to stratify by disease stage, the predominantly cross-sectional design of the included studies with relatively limited power, inconsistency of the depression and anxiety assessment method, and considerable differences in the prevalence estimates across the included studies owing to the high levels of heterogeneity identified for both depression and anxiety. Additionally, a population-based cohort study from Taiwan failed to identify a relationship between anxiety disorder and the risk of cancer [36].

Furthermore, several studies have shown that ER, PR, and p53 expression were the independent prognostic factors for survival in OC patients [37,38], whereas vimentin expression was positively correlated with histological grade of serous OC [39]. WT-1 was a highly sensitive and specific IHC marker for diagnosing ovarian high-grade serous carcinomas [40]. It has been indicated that serous OC patients have better survival compared with other histological types in previous studies of OOPS [17]. What is more, our findings supported the view that the association between depression and OC survival might be modified by immunohistochemical biomarkers, such as WT-1. However, as the number of patients in some categories was relatively limited, we could not rule out the possibility of chance findings, and future studies are warranted to validate these interactions.

Several underlying mechanisms may be biological in OC patients with depression. Depression may be expected to result in abnormal activation of the hypothalamic–pituitary–adrenal axis and high norepinephrine and cortisol levels, with consequent suppression of the immune response to tumors [8]. After that, long-term dysfunction of the immune system might give rise to the suppression of DNA-repair enzymes and NK cell function, leading to a worse prognosis [8,41]. Furthermore, evidence shows that depression is related to inflammatory markers such as interleukin 1,6 and C-reactive protein, which could be used as independent prognostic variables of OC [42,43]. An animal experiment has validated that chronic stress can result in higher levels of tissue catecholamines, greater tumor burden, and more invasive growth of OC [44]. Mounting evidence has highlighted the substantial genetic, neurobiological, and symptomatic overlap between depression and anxiety disorders that frequently co-occur [45,46,47]. Furthermore, previous evidence suggested that anxiety disorder was associated with shorter telomeres [48] and higher circulating levels of inflammatory cytokines [49], both related to OC risk and progression [50,51,52]. Future studies should further explore the exact and detailed biological mechanisms of depression and anxiety symptoms and their influence on the prognosis of OC.

Notably, some explanations might be related to psychological behavior changes among OC patients with depression. For example, individuals with depression may be more prone to unhealthy lifestyles, including physical inactivity, malnutrition, and insomnia, resulting in diminished quality of life and a reduction in the ability to care for oneself. [53,54,55]. Treatment nonadherence also might be a severe problem. There is evidence that cancer patients with depression symptom have difficulties adhering to chemotherapy and other complementary treatments, which could lead to faster cancer progression [8,56,57,58]. In addition, depression was highly correlated with demoralization characterized by hopelessness, helplessness, and loss of meaning and purpose, which was associated with suicidal thoughts and the desire to die [59,60,61]. A mediation analysis has shown that hopelessness influenced the desire for hastened death, whereas depression was a moderator in the relationship between them [62]. Moreover, depression rendered patients less capable of functioning successfully in society and diminished purpose in life, and thus could contribute to worse OC survival [63,64].

## 6. Strengths and Limitations

Our study has several unique strengths. Firstly, we investigated the association of prediagnosis depression and anxiety symptoms and their combined symptoms and OC survival in a prospective cohort study. Additionally, our study used the validated PHQ-9 and GAD-7 to assess depression and anxiety symptoms, respectively. Reliable related measures were obtained by trained staff and confirmation of death events through medical records and cancer registry. Besides, we validated varying information on a broad spectrum of known or plausible confounders, such as lifestyle factors, dietary habits, BMI, sleep duration and quality, and physical activity, which allowed relatively rigorous control for confounding throughout the analysis to minimize potential bias. Notably, the OOPS had excellent baseline and follow-up retention rates (92.7%). Finally, the numerous subgroup analyses that we performed generated similar results, indicating that our findings were robust.

However, several limitations of this study should be noted. Firstly, information on the dynamic duration of depression, anxiety symptoms, history of depression and anxiety, and the antidepressant used was not available. Secondly, since depression and anxiety symptoms of patients were measured within the year prior to diagnosis, we were concerned that undetected OC might cause mood swings during the data collection period. Furthermore, the prediagnosis data may not reflect postdiagnosis status. A previous study revealed that the level of depression and anxiety varies significantly between pre- and postdiagnosis [11].

Given that the mental condition of some patients will change after a cancer diagnosis, future studies are needed to investigate the relationship between postdiagnosis depression and anxiety symptoms or possible change between pre- and postdiagnosis psychiatric symptoms and OC survival. Thirdly, the relationship between depression and anxiety and mortality is very much driven by the symptom characteristics—severity, chronicity, and type [65]. In this study, severely depressed women were less likely to volunteer to participate, and the number of patients with depression and anxiety symptoms was small, meaning that there was insufficient statistical power for detailed further analyses. Moreover, all patients were recruited from a single referral hospital in northeast China, making it difficult to generalize the results. Given that differing rates of depression and anxiety have emerged between racial and ethnic groups [66,67], more oncology centers should be established in the various regions. We used all-cause mortality rather than cancer-specific mortality as an end point, which might reduce the power in our study, although the former is considered a reasonable estimate of the latter [68]. Further research should consider more outcomes. Lastly, despite comprehensively adjusting for several established or hypothesized cancer prognostic and lifestyle factors for OC, there is always the possibility that unrecognized residual confounding factors or errors may have influenced the results shown or that some findings were due to chance. Individuals with depression and anxiety might be more prone to unhealthy lifestyles, delayed cancer diagnosis, and treatment nonadherence, which could indirectly increase the risk of mortality [53,58].

In conclusion, in this prospective study, an elevated risk of OC mortality was associated with prediagnosis depression, but not anxiety symptoms. However, further large multicenter studies are warranted to validate our findings and better understand the effect of depression and anxiety symptoms on OC survival.

## Figures and Tables

**Table 1:** Baseline characteristics of ovarian cancer patients by depression and anxiety symptoms (*n* = 598).

Characteristics	All Patients	Depression Symptoms ^†^		Anxiety Symptoms ^‡^	
		Yes	No	Yes	No
No. of patients/deaths	598/130	56/19	542/111	235/49	363/81
Mean (SD) age at diagnosis (years)	53.34 (9.45)	53.68 (7.40)	53.30 (9.64)	53.04 (9.21)	53.53 (9.61)
Mean (SD) follow-up time (months)	35.01 (16.86)	32.08 (19.19)	35.31 (16.60)	35.57 (15.01)	34.64 (17.97)
Mean (SD) body mass index (kg/m^2^)	23.25 (3.55)	22.64 (3.50)	23.31 (3.55)	23.41 (3.52)	23.15 (3.57)
Mean (SD) physical activity (MET hours/day)	15.80 (11.11)	15.96 (12.54)	15.78 (10.97)	16.34 (11.43)	15.45 (10.90)
Mean (SD) sleep duration (hours) *	7.73 (1.32)	7.42 (1.47)	7.77 (1.30)	7.48 (1.30)	7.88 (1.32)
Mean (SD) sleep quality *	6.35 (3.95)	9.94 (3.96)	6.00 (3.78)	7.09 (3.98)	5.86 (3.86)
Ever cigarette smoking	64 (10.70)	3 (5.36)	61 (11.25)	26 (11.06)	38 (10.47)
Ever alcohol drinking	135 (22.58)	13 (23.21)	122 (22.51)	52 (22.13)	83 (22.87)
Ever tea drinking	195 (32.61)	15 (26.79)	180 (33.21)	73 (31.06)	122 (33.61)
Ever menopause	435 (72.74)	41 (73.21)	394 (72.69)	161 (68.51)	274 (75.48)
Experienced stressful life events in past two years	218 (36.45)	27 (48.21)	191 (35.24)	90 (38.30)	128 (35.26)
Ever dietary change before diagnosis	136 (22.74)	10 (17.86)	126 (23.25)	56 (23.83)	80 (22.04)
Parity					
≤1	421 (70.40)	37 (66.07)	384 (70.85)	162 (68.94)	259 (71.35)
≥2	177 (29.60)	19 (33.93)	158 (29.15)	73 (31.06)	104 (28.65)
Educational level					
Junior secondary or below	315 (52.68)	38 (67.86)	277 (51.11)	123 (52.34)	192 (52.89)
Senior high school/technical secondary school	125 (20.90)	9 (16.07)	116 (21.40)	47 (20.00)	78 (21.49)
Junior college/university or above	158 (26.42)	9 (16.07)	149 (27.49)	65 (27.66)	93 (25.62)
Income per month (Yuan)					
<5000	357 (59.70)	37 (66.07)	320 (59.04)	128 (54.47)	229 (63.09)
5000 to <10,000	167 (27.93)	16 (28.57)	151 (27.86)	66 (28.09)	101 27.82)
≥10,000	74 (12.37)	3 (5.36)	71 (13.10)	41 (17.45)	33 (9.09)
Mean (SD) total energy intake (kcal/day)	1457.96 (565.15)	1390.86 (487.45)	1464.89 (572.53)	1466.96 (583.19)	1452.13 (553.90)
Mean (SD) vegetables intake (gram/day)	217.99 (123.71)	213.04 (102.80)	218.50 (125.74)	212.28 (117.05)	221.69 (127.86)
Mean (SD) fruit intake (gram/day)	197.80 (155.14)	189.50 (129.85)	198.66 (157.60)	205.68 (164.59)	192.70 (148.71)
Mean (SD) meat intake (gram/day)	36.86 (29.96)	38.42 (26.90)	36.70 (30.27)	38.89 (31.82)	35.55 (28.65)
Mean (SD) soy food intake (gram/day)	87.08 (80.63)	81.65 (65.39)	87.65 (82.08)	83.63 (78.69)	89.32 (81.90)

MET, metabolic equivalents of task; SD, standard deviation. * Sleep duration and quality was evaluated according to Pittsburgh sleep quality index. ^†^ Depression symptom was evaluated according to Patient Health Questionnaire-9 (No: score <10). ^‡^ Anxiety symptom was evaluated according to Generalized Anxiety Disorder-7 (No: score <5). Values are numbers (percentages) unless stated otherwise.

**Table 2:** Adjusted hazard ratio and 95% confidence interval for the interaction between depression and anxiety symptoms and total mortality among 598 ovarian cancer patients *.

	Anxiety Symptoms			P Interaction ^†^
	None (Score <5)	Moderate (Score 5–9)	Severe (Score ≥10)	
Depression symptoms				0.26
None (score <10)	1.00 (Ref.)	0.64 (0.36–1.15)	2.23 (0.63–8.39)	
Moderate (score 10–14)	1.32 (0.29–5.93)	3.23 (1.14–9.11)	—	
Severe (score ≥15)	2.52 (0.69–9.26)	2.28 (0.75–6.95)	1.50 (0.51–4.39)	

Ref., reference. Depression symptom was evaluated according to Patient Health Questionnaire-9. Anxiety symptom was evaluated according to Generalized Anxiety Disorder-7. * Hazard ratio and 95% confidence interval was calculated with the use of the Cox proportional hazards regression model. ^†^ Test for interaction based on depression and anxiety symptoms. Model was adjusted for age at diagnosis, body mass index, income level, dietary pattern, sleep duration and quality, dietary change, education, number of menstrual years, parity, physical activity, experience any major events in the past two years, comorbidities, FIGO stage, histological type, histopathologic grade, and residual lesions.

## Data Availability

Data are available upon request from the corresponding author.

## Supplementary Materials

The following supporting information can be downloaded at: https://www.mdpi.com/article/10.3390/jcm11247394/s1, Table S1. Selected clinical characteristics and associations with overall survival among ovarian cancer patients (*n* = 598). Table S2. Adjusted hazard ratio and 95% confidence intervals for the association between depression and anxiety symptoms and total mortality among 598 ovarian cancer patients *. Figure S1. Kaplan–Meier survival curves for depression symptom (A), anxiety symptom (B). Figure S2. Subgroup analyses of clinical characteristics for adjusted hazard ratio and 95% confidence interval for the association between depression symptom and total mortality among 598 ovarian cancer patients. Figure S3. Subgroup analyses of clinical characteristics for adjusted hazard ratio and 95% confidence interval for the association between anxiety symptom and total mortality among 598 ovarian cancer patients. Figure S4. Subgroup analyses of immunohistochemical biomarkers for adjusted hazard ratio and 95% confidence interval for the association between depression symptom and total mortality among 598 ovarian cancer patients. Figure S5. Subgroup analyses of immunohistochemical biomarkers for adjusted hazard ratio (HR) and 95% confidence interval (CI) for the association of anxiety symptom with total mortality among ovarian cancer patients.
