# Supplementary material for: Prediagnosis Depression Rather Than Anxiety Symptoms Is Associated with Decreased Ovarian Cancer Survival: Findings from the Ovarian Cancer Follow-Up Study (OOPS)
Source: J Clin Med. 2022 Dec 13;11(24):7394. doi: 10.3390/jcm11247394 (PMC9781310; doi:10.3390/jcm11247394)
Supplement: Supplementary file 1 [file jcm-11-07394-s001.zip › jcm-2051891-supplementary.pdf]

Supplementary Table S1. Selected clinical characteristics and associations with overall survival among ovarian cancer patients ($n = 598$).

Characteristics	No. of Deaths/Total (%)	Adjusted HR * (95%CI)
**Age at diagnosis**		
≤50	45/224 (34.62)	1.00 (Ref)
>50	85/374 (65.38)	1.29 (0.89–1.87)
**Histological type**		
Serous	92/409 (70.77)	1.00 (Ref)
Non-serous	38/189 (29.23)	1.75 (1.13–2.71)
**Histopathologic grade**		
Well differentiated	5/45 (3.85)	1.00 (Ref)
Moderately differentiated	7/42 (5.38)	1.73 (0.68–4.37)
Poorly differentiated	118/511 (90.77)	1.07 (0.34–3.43)
**FIGO stage**		
I–II	41/305 (31.54)	1.00 (Ref)
III–IV	89/284 (68.46)	2.64 (1.72–4.05)
**Residual lesions**		
No	82/469 (63.08)	1.00 (Ref)
<1 cm	31/90 (23.85)	1.72 (1.11–2.66)
≥1 cm	17/39 (13.08)	2.40 (1.39–4.16)
**Comorbidities**		
No	74/313 (56.92)	1.00 (Ref)
Yes	56/285 (43.08)	0.89 (0.62–1.27)

CI, confidence interval; HR, hazard ratio; Ref, reference. * Mutually adjusted for all other variables listed in the table.

Supplementary Table S2. Adjusted hazard ratio and 95% confidence intervals for the association between depression and anxiety symptoms and total mortality among 598 ovarian cancer patients *.

Characteristics	Deaths, N (% of Total Deaths)	Model 1 HR (95% CI)	Model 2 HR (95% CI)	Model 3 HR (95% CI)
**Depression**				
No (score <10)	111 (85.38)	1.00 (Ref)	1.00 (Ref)	1.00 (Ref)
Yes (score ≥10)	19 (14.62)	1.88 (1.15–3.06)	2.11 (1.20–3.70)	2.10 (1.20–3.70)
**Severity of Depression**				
Moderate (score 10–14)	8 (6.15)	1.75 (0.85–3.61)	2.02 (0.88–4.63)	2.11 (0.88–5.07)
Severe (score ≥15)	11 (8.46)	1.97 (1.06–3.67)	2.17 (1.11–4.21)	2.10 (1.07–4.12)
**Anxiety**				
No (score <5)	81 (62.31)	1.00 (Ref)	1.00 (Ref)	1.00 (Ref)
Yes (score ≥5)	49 (37.69)	0.92 (0.65–1.32)	0.89 (0.57–1.40)	0.94 (0.60–1.48)
**Severity of Anxiety**				
Moderate (score 5–9)	41 (31.54)	0.84 (0.58–1.23)	0.79 (0.49–1.28)	0.85 (0.52–1.40)
Severe (score ≥10)	8 (6.15)	1.79 (0.86–3.70)	1.68 (0.72–3.91)	1.46 (0.62–3.45)

CI, confidence interval; HR, hazard ratio; Ref, reference; SD, standard deviation. Depression symptom was evaluated according to Patient Health Questionnaire-9. Anxiety symptom was evaluated according to Generalized Anxiety Disorder-7. * HR and 95% CI were calculated with the use of the Cox proportional hazards regression model. Model 1 was adjusted for age at diagnosis and body mass index. Model 2 was further adjusted for income level, dietary pattern, sleep duration and quality, dietary change, education, number of menstrual years, parity, physical activity, and experience any major events in the past two years based on Model 1. Model 3 was further adjusted for comorbidities, FIGO stage, histological type, histopathologic grade, and residual lesions based on Model 2.

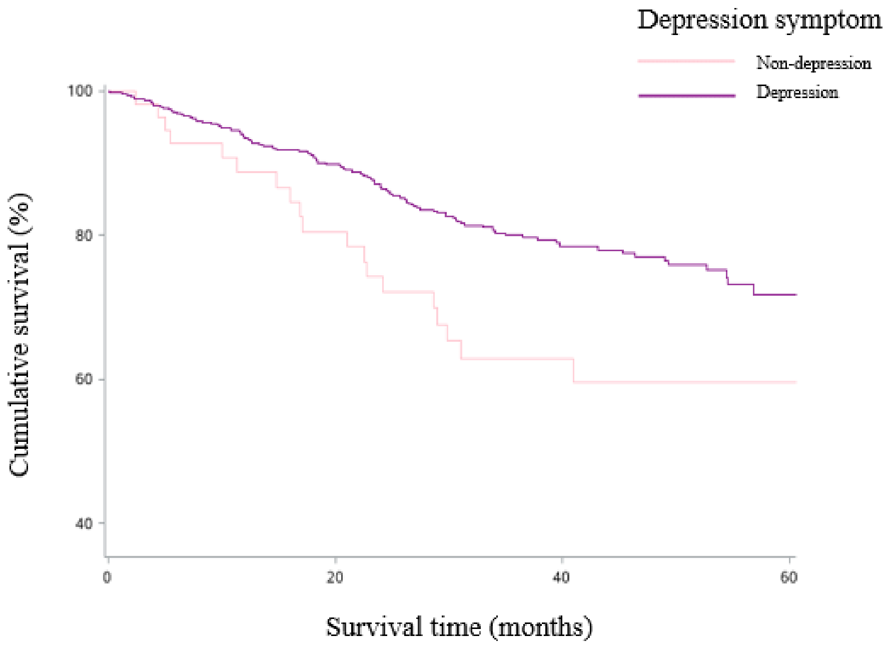
Depression symptom
Non-depression
Depression
Cumulative survival (%)
100
80
60
40
0
20
40
60
Survival time (months)

(A)

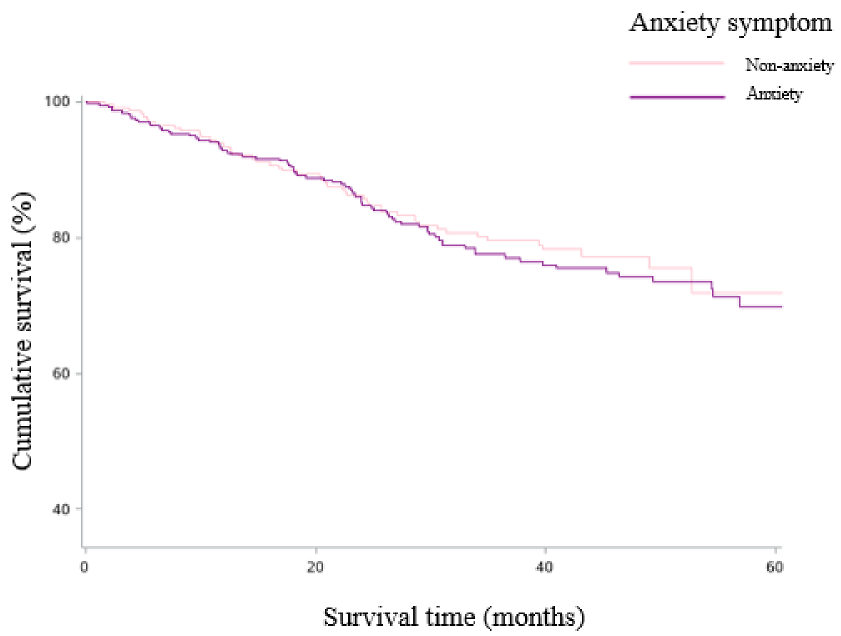

(B)

Supplementary Figure S1. Kaplan–Meier survival curves for depression symptom (A), anxiety symptom (B).

Depression symptoms †

Supgroup	Harzard Ratio	95% CI	$P_{interaction}$ *
**Age at diagnosis (years)**			< 0.05
≤ 50	7.78	2.65 - 22.86	
> 50	1.29	0.62 - 2.65	
**Menopausal status**			0.23
No	2.73	0.81 - 9.22	
Yes	1.74	0.84 - 3.62	
**Body mass index (kg/m^2)**			0.26
< 24	2.91	1.30 - 6.51	
≥ 24	1.73	0.71 - 4.21	
**Sleep duration (hours/day) ****			0.48
< 8	1.07	0.50 - 2.28	
≥ 8	2.6	1.06 - 6.40	
**Sleep quality (score) ****			0.53
< 5	1.97	0.65 - 5.94	
≥ 5	2.15	1.07 - 4.33	
**Experienced stressful life events**			0.34
Yes	0.86	0.34 - 2.16	
No	2.31	1.03 - 5.21	
**Histological type**			< 0.05
Serous	1.2	0.52 - 2.74	
Non-serous	8.52	2.61 - 27.82	
**FIGO stage**			1
I-II	4.87	1.68 - 14.81	
III-IV	1.37	0.64 - 2.93	
**Residual lesions**			0.2
No	1.51	0.73 - 3.10	
Yes	4.93	1.66 - 14.68	

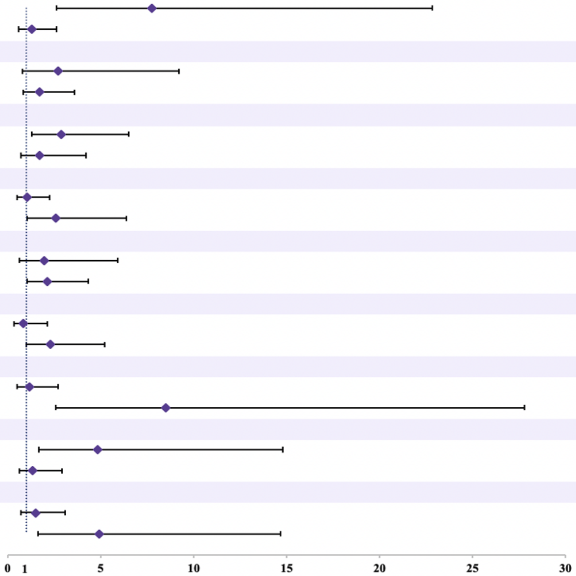

Supplementary Figure S2. Subgroup analyses of clinical characteristics for adjusted hazard ratio and 95% confidence interval for the association between depression symptom and total mortality among 598 ovarian cancer patients. * Test for interaction based on strata and depression symptom. ** Sleep duration and quality was evaluated according to Pittsburgh sleep quality index. † Depression symptom was evaluated according to Patient Health Questionnaire-9 (No: score < 10). Hazard ratio and 95% confidence interval was calculated with the use of the Cox proportional hazards regression model with age at diagnosis, body mass index, income level, dietary pattern, sleep duration and quality, dietary change, education, number of menstrual years, parity, physical activity, experience any major events in the past two years, comorbidities, FIGO stage, histological type, histopathologic grade, and residual lesions.

Anxiety symptoms ‡

Supgroup	Harzard Ratio	95% CI	$P_{interaction}$ *
**Age at diagnosis (years)**			0.19
≤ 50	4.47	0.95 - 20.95	
> 50	0.94	0.31 - 2.82	
**Menopausal status**			0.16
No	4.09	0.73 - 22.89	
Yes	1	0.33 - 3.05	
**Body mass index (kg/m^2)**			0.68
< 24	1.43	0.45 - 4.52	
≥ 24	2.32	0.53 - 10.08	
**Sleep duration (hours/day) ****			0.91
< 8	1.74	0.60 - 5.09	
≥ 8	1.28	0.37 - 4.47	
**Sleep quality (score) ****			0.38
< 5	1.42	0.35 - 5.77	
≥ 5	1.56	0.47 - 5.15	
**Experienced stressful life events**			0.96
Yes	0.28	0.06 - 1.21	
No	3.84	1.20 - 12.31	
**Histological type**			0.06
Serous	1.08	0.38 - 3.06	
Non-serous	1.43	0.21 - 9.87	
**FIGO stage**			1.00
I-II	1.11	0.90 - 1.94	
III-IV	2.2	0.86 - 5.61	
**Residual lesions**			0.24
No	0.62	0.16 - 2.39	
Yes	2.99	0.84 - 10.63	

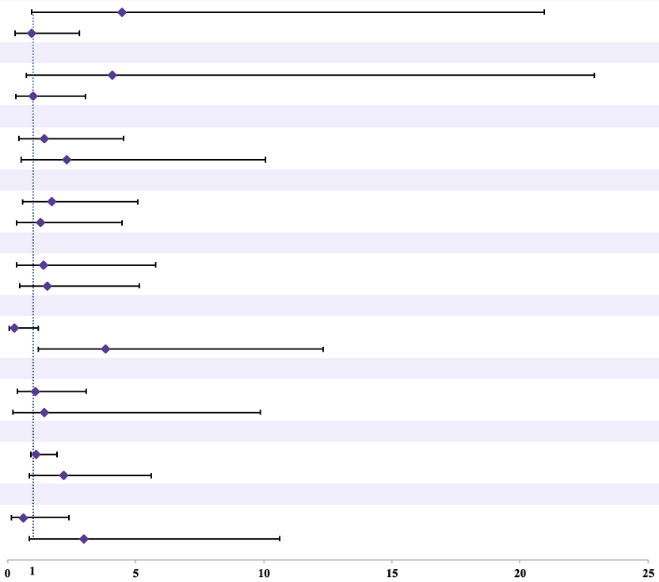

Supplementary Figure S3. Subgroup analyses of clinical characteristics for adjusted hazard ratio and 95% confidence interval for the association between anxiety symptom and total mortality among 598 ovarian cancer patients. * Test for interaction based on strata and anxiety symptom. ** Sleep duration and quality was evaluated according to Pittsburgh sleep quality index. ‡ Anxiety symptom was evaluated according to Generalized Anxiety Disorder-7. Hazard ratio and 95% confidence interval was calculated with the use of the Cox proportional hazards regression model with age at diagnosis, body mass index, income level, dietary pattern, sleep duration and quality, dietary change, education, number of menstrual years, parity, physical activity, experience any major events in the past two years, comorbidities, FIGO stage, histological type, histopathologic grade, and residual lesions.

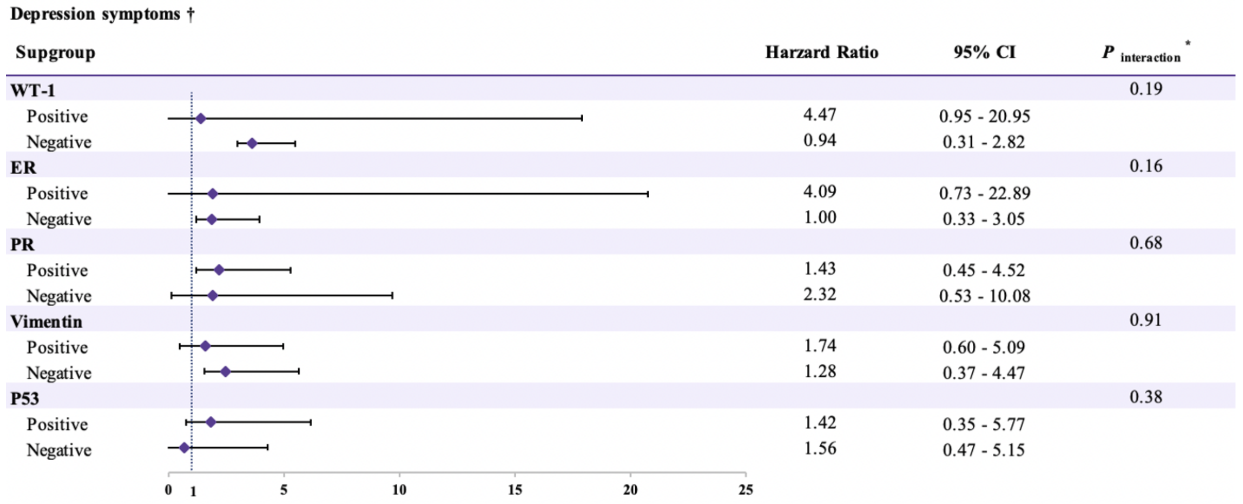

Supplementary Figure S4. Subgroup analyses of immunohistochemical biomarkers for adjusted hazard ratio and 95% confidence interval for the association between depression symptom and total mortality among 598 ovarian cancer patients. * Test for interaction based on strata and depression symptom. ** Sleep duration and quality was evaluated according to Pittsburgh sleep quality index. † Depression symptom was evaluated according to Patient Health Questionnaire-9 (No: score < 10). Hazard ratio and 95% confidence interval was calculated with the use of the Cox proportional hazards regression model with age at diagnosis, body mass index, income level, dietary pattern, sleep duration and quality, dietary change, education, number of menstrual years, parity, physical activity, experience any major events in the past two years, comorbidities, FIGO stage, histological type, histopathologic grade, and residual lesions.

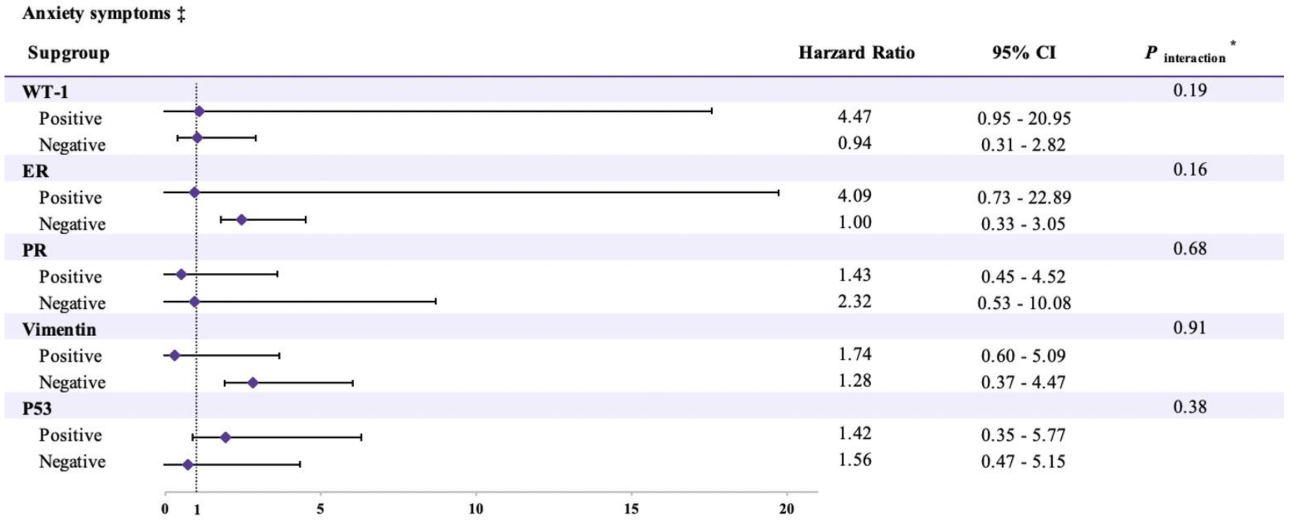

Supplementary Figure S5. Subgroup analyses of immunohistochemical biomarkers for adjusted hazard ratio (HR) and 95% confidence interval (CI) for the association of anxiety symptom with total mortality among ovarian cancer patients. * Test for interaction based on strata and anxiety symptom. ** Sleep duration and quality was evaluated according to Pittsburgh sleep quality index. ‡ Anxiety symptom was evaluated according to Generalized Anxiety Disorder-7. Hazard ratio and 95% confidence interval was calculated with the use of the Cox proportional hazards regression model with age at diagnosis, body mass index, income level, dietary pattern, sleep duration and quality, dietary change, education, number of menstrual years, parity, physical activity, experience any major events in the past two years, comorbidities, FIGO stage, histological type, histopathologic grade, and residual lesions.
